# Angiotensin II for the treatment of vasodilatory shock: enough data to consider angiotensin II safe?

**DOI:** 10.1186/s13054-018-2006-0

**Published:** 2018-04-16

**Authors:** Nina Buchtele, Michael Schwameis, Bernd Jilma

**Affiliations:** 1 Department of Clinical Pharmacology, Medical University of Vienna, Waehringer Guertel 18-20, A-1090 Vienna, Austria; 2 Department of Emergency Medicine, Medical University of Vienna, Waehringer Guertel 18-20, A-1090 Vienna, Austria

A recent structured review by Busse et al. confirmed the efficacy of angiotensin II in terms of increasing blood pressure in patients with different types of shock [1]. The majority of data for this analysis came from the recently published ATHOS-3 trial which showed that angiotensin II effectively increased blood pressure in patients with vasodilatory shock receiving high-dose vasopressors [2]. In this trial, safety was defined as the secondary outcome. Considering the similar total number of adverse events in both groups, the administration of angiotensin II seemed safe. Antonucci et al. had already raised some caution about the administration of angiotensin II [3]. In addition, we want to add some further comments about the use of angiotensin II, especially as it has recently been approved by the US Food and Drug administration.

Taking a closer look at the subgroups of adverse events, patients in the treatment group were significantly more likely to develop infection or delirium. High doses of vasopressors are known to cause mesenteric ischemia [4], which may impair intestinal mucosal barrier function resulting in transmission of enteric bacteria into the bloodstream. It would be of great interest to analyse these specific adverse events in another structured review or meta-analysis.

Furthermore, the significant decrease in the cardiovascular Sequential Organ Failure Assessment (SOFA) score in the treatment group must be interpreted cautiously. The infusion of angiotensin II probably allowed a decrease in background catecholamines and therefore a decrease in the cardiovascular SOFA score. The SOFA score accurately predicts mortality of critically ill patients, but the cardiovascular domain has its limitations [5]. It is unclear whether the cardiovascular component of the SOFA score can be used if vasopressors other than catecholamines are infused. The key issue is whether additional points for equivalent doses of angiotensin have to be included in the score. Although hypotension improved, the total score worsened to a similar extent in both groups, suggesting that functions of other organ systems may have deteriorated.

It seems that finally efforts in developing a new effective substance in refractory shock have paid off, but the safety results have to be treated with caution.
