# Supplementary material for: Angiotensin II for the treatment of vasodilatory shock: enough data to consider angiotensin II safe?
Source: Crit Care. 2018 Apr 16;22:96. doi: 10.1186/s13054-018-2006-0 (PMC5902841; doi:10.1186/s13054-018-2006-0)
Supplement: Related texts

# Reply: Authors’ response

**Authors:** Laurence W. Busse, Mizra Haider Ali

It has recently been re-established that angiotensin II is effective in raising blood pressure in patients with hypotension [1, 2]. Dr. Buchtele and colleagues raise the subject of the safety of angiotensin II, which was evaluated as a secondary outcome in ATHOS-3. While there were adverse event imbalances between the angiotensin II and placebo groups, the total incidence was similar. More importantly, the safety of angiotensin II was recently examined in a comprehensive review of over 31,000 subjects and was shown to be well tolerated, with only two deaths and fewer than 300 subjects with adverse events reported [6]. Nonetheless, we agree that further investigation should focus on the imbalances noted in ATHOS-3 regarding safety, including delirium, infection, and thromboembolism.

The use of the cardiovascular SOFA score as a secondary endpoint in ATHOS-3 must indeed be understood cautiously. Dr. Buchtele and colleagues rightly point out that the infusion of angiotensin II allowed a decrease in the background catecholamines resulting in a decrease in the score. This was, in fact, the sole purpose of this end-point, which was never intended to be interpreted clinically. The cardiovascular SOFA score allowed for standardisation of different vasopressor regimens specifically to observe the sparing effect that angiotensin II had on norepinephrine equivalent vasopressors. The norepinephrine equivalence of phenylephrine and vasopressin, not included in SOFA, are best approximations of equivalence based on available medical literature, but specific equivalence conversions are not important. Rather, ATHOS-3 showed that, whatever the vasopressor cocktail, angiotensin II allowed for sparing of catecholamines, which are known to be associated with mortality and adverse events [7, 8]. The message elucidated by this end-point is that a balanced approach, using lower doses of vasopressors with different mechanisms of action, effectively raises blood pressure and may avoid some of the toxicities associated with high doses of single agent therapy.

Finally, just as the cardiovascular SOFA should be interpreted with caution, so too should the total SOFA score. While total SOFA score increased in both the placebo group and the angiotensin II group in ATHOS-3 (with no differences in this increase between groups), this did not translate into increased mortality for both groups; in fact, there was a trend toward improvement in survival in the angiotensin II group.

We agree with Dr. Buchtele and colleagues that the safety profile of angiotensin II should be scrutinized thoroughly, as with all novel therapies. That said, the addition of another tool in the clinician’s tool box for the treatment of circulatory shock is, in our opinion, a welcome development.
